# Application of Simultaneous and Coupled Thermal Analysis Techniques in Studies on the Melting Process, Course of Pyrolysis and Oxidative Decomposition of Fused Triazinylacetohydrazides

**DOI:** 10.3390/ijms25020813

**Published:** 2024-01-09

**Authors:** Marta Worzakowska, Krzysztof Sztanke, Małgorzata Sztanke

**Affiliations:** 1 Department of Polymer Chemistry, Institute of Chemical Sciences, Faculty of Chemistry, Maria Curie-Sklodowska University in Lublin, 33 Gliniana Street, 20-614 Lublin, Poland; marta.worzakowska@mail.umcs.pl; 2 Laboratory of Bioorganic Compounds Synthesis and Analysis, Medical University of Lublin, 4A Chodźki Street, 20-093 Lublin, Poland; 3 Department of Medical Chemistry, Medical University of Lublin, 4A Chodźki Street, 20-093 Lublin, Poland; malgorzata.sztanke@umlub.pl

**Keywords:** DSC, TG/DTG/FTIR/QMS methods, thermal studies, thermal degradation mechanism, thermal decomposition course, annelated triazinylacetohydrazides, heterocyclic hydrazides, polyazaheterocycles, antioxidant agents

## Abstract

The effect of the structure of promising antioxidant agents with prospective medical use, i.e., unsubstituted and *para*-substituted annelated triazinylacetic acid hydrazides, on their melting points, thermal stabilities, pyrolysis and oxidative decomposition stages and the type of volatiles emitted under heating with the use of DSC and TG/DTG/FTIR/QMS methods was evaluated and discussed. The melting point of the investigated compounds increased with an enhanced number of electrons (directly correlated with their molecular weight). Melting enthalpy values were determined and presented for all the studied compounds. The pyrolysis and oxidative decomposition processes of the analysed molecules consisted of several poorly separated stages, which indicated a multi-step course of the decomposition reactions. It was found that the thermal stability of the tested compounds depended on the type of substituent at the *para* position of the phenyl moiety or its absence. In both atmospheres used (air and helium), the thermal stability increased in relation to R as follows: -CH_3_ ≤ -OCH_3_ < -H < -OC_2_H_5_. In an inert atmosphere, it was higher by approx. 8–18 °C than in an oxidative atmosphere. The pyrolysis was connected with the emission of NH_3_, HCN, HNCO, HCONH_2_, HCHO, CO_2_, CO and H_2_O in the case of all the tested compounds, regardless of the substituent attached. In the case of the derivative containing the *para*-CH_3_ group, *para*-toluidine was an additional emitted aromatic product. In turn, emissions of aniline and alcohol (methanol or ethanol) for compounds with the *para*-OCH_3_ and *para*-OC_2_H_5_ groups, respectively, were confirmed. In oxidative conditions, the release of NH_3_, NO, HCN, HNCO, HCONH_2_, CO_2_, H_2_O and cyanogen (for all the compounds) and *para*-toluidine (for the *para*-CH_3_ derivative), aniline (for *para*-OCH_3_, *para*-OC_2_H_5_ and unsubstituted derivatives) and acetaldehyde (for the *para*-OC_2_H_5_ derivative) were clearly observed. No alcohol emissions were recorded for either compound containing the *para*-OCH_3_- or *para*-OC_2_H_5_-substitututed phenyl ring. These results confirmed that the pyrolysis and oxidative decomposition of the investigated annelated triazinylacetohydrazides occurred according to the radical mechanism. Moreover, in the presence of oxygen, the reactions of volatiles and residues with oxygen (oxidation) and the combustion process additionally proceeded.

## 1. Introduction

**Figure 1:**
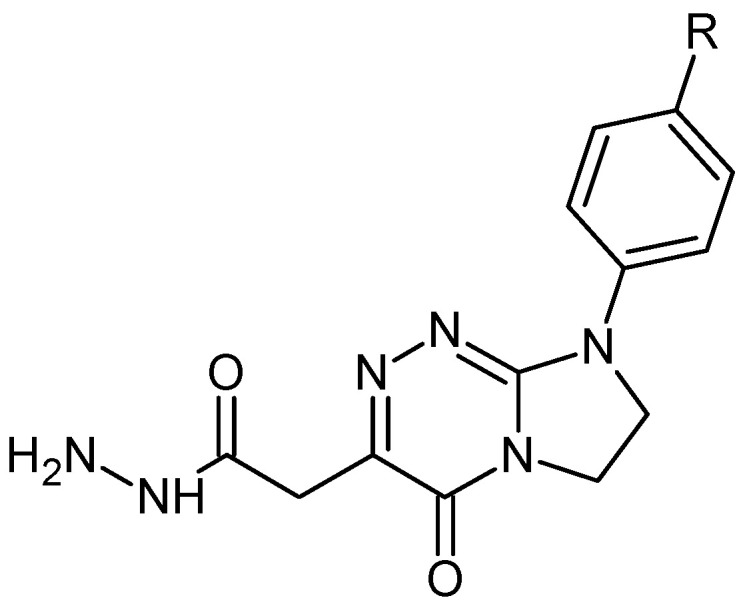
The general structure of polynitrogenated annelated triazines with the common acetohydrazide functional group. The set subjected to thermal studies includes the parent compound and its *para*-substituted derivatives: **1**: R = H; **2**: R = CH_3_; **3**: R = OCH_3_; **4**: R = OC_2_H_5_.

Fused azaisocytosine-containing congeners with the acetohydrazide functional group (**1**–**4**, Figure 1), which is prone to quick oxidation, are an important class of nitrogen-rich heterocycles. All these annelated triazinylacetic acid hydrazides, including the parent compound (**1**, as an unsubstituted structure) and the remaining *para*-substituted derivatives (**2**–**4**), have been synthesised formerly, and their spectroscopic data were consistent with the assigned structures shown in Figure 1 [1]. These compounds have previously been subjected to extensive biological assays evaluating their reducing power and antioxidant potential, as a result of which some of them have been disclosed as new, promising antioxidant agents with prospective utility in the treatment of diseases induced by reactive oxygen species and non-radical reactive oxygen metabolites. In this class of substituted annelated triazines with the common acetohydrazide functional group, the methoxy derivative (**3**) has previously been reported as the most promising antioxidant agent candidate because its ability to neutralise free radicals (such as DPPH^•^—2,2-diphenyl-1-picrylhydrazyl radical and nitric oxide radical) and non-radical reactive oxygen metabolites (such as hydrogen peroxide) is superior or similar to that of the antioxidants in common use. Moreover, the strongest scavenger of hydrogen peroxide proved to be the ethoxy derivative (**4**), whose effect is better than that of standard antioxidants. For all the molecules bearing the common acetohydrazide pharmacophore (**1**–**4**), the mechanism of their antiradical action (through hydrogen atom transfer to the DPPH^•^) has already been established [1]. This makes them promising drug candidates in the preclinical phase of drug development. Such synthetic annelated triazinylacetetohydrazides may be of benefit in the prevention/therapy of various oxidative-stress-mediated diseases.

There is a number of known drugs and potential molecular pharmaceutics that contain the privileged scaffold of 1,2,4-triazine or 1,2,4-triazinone [2,3,4,5]. Nevertheless, there are only few scientific reports on the thermal analysis of drugs structurally based on the 1,2,4-triazine system (lamotrigine—used for the treatment of epilepsy and bipolar disorder) [6,7] or with the 1,2,4-triazin-4(3*H*)-one nucleus incorporated into the bicyclic structure (vardenafil—used for the treatment of erectile dysfunction) [8]. However, surveys have shown that the thermal stability and decomposition studies have been carried out on many condensed 1,2,4-triazines containing some functional moieties, including those with importance in material science [9] and pharmaceutical science [10,11,12]. On the other hand, there is a limited number of scientific reports on the thermal analysis of small molecules containing the acetohydrazide functional group. These papers concern 2-(1*H*-indol-3-yl)acetohydrazide, 2-[(4-bromophenyl)amino]acetohydrazide, 2-(1*H*-benzotriazol-1-yl)acetohydrazide and 2-(4,6-dimethyl-1*H*-pyrazolo[3,4-*b*]pyridine-1-yl)acetohydrazide) [13,14,15,16].

However, there is a research gap in the thermal studies performed. Nothing is known about the thermal properties or the mechanism and course of thermal degradation of an important class of annelated triazinylacetic acid hydrazides, i.e., 2-[4-oxo-8-(R-phenyl)-4,6,7,8-tetrahydroimidazo[2,1-*c*][1,2,4]triazin-3-yl]acetohydrazides, that may have potential in the prevention of free-radical-mediated diseases. Therefore, in the preclinical phase of drug development, the detailed thermal assessment of these molecules is needed. Knowledge about the behaviour during heating of annelated triazinylacetohydrazides, so far unknown, is essential for their further characterisation, as well as for determining the conditions necessary during their storage and processing.

The present study was carried out with the goal of characterising, for the first time, the detailed thermal behaviour of this class of pharmacologically important heterobicyclic acetic acid hydrazides, employing thermal techniques such as differential scanning calorimetry (DSC) and thermogravimetric analysis (TG/DTG) coupled with Fourier-transform infrared spectroscopy and quadrupole mass spectrometry (FTIR/QMS) analysers. Simultaneous thermal analysis methods coupled with both spectroscopic and spectrometric techniques enable the reliable interpretation of the thermal behaviour and understanding of the degradation pathway of pharmaceutical substances and potential drugs [17]. Therefore, simultaneous and coupled thermal techniques are recommended and commonly used in thermal testing of molecular pharmaceutics and potential drug candidates. Additionally, thermal analysis technologies—which have been reviewed—are commonly applied for studying the thermal stability and thermal properties of energetic materials [18].

The novelty of the present investigation is assessing the thermal stability of annelated triazinylacetohydrazides under inert and oxidative conditions and describing the thermal effects (characterised by a temperature range and enthalpy change) of all the analysed unsubstituted and *para*-substituted molecules, as well as identifying the volatile decomposition products emitted during their pyrolysis in helium and oxidative decomposition in air. We performed the TG/DTG/FTIR/QMS analyses under inert and oxidative conditions with the subsequent aim of elucidating the thermal decomposition mechanism and course of these heterobicyclic molecules with the common acetohydrazide functional moiety and identifying the possible degradation products in relation to the molecular structure of the particular derivative undergoing pyrolysis. In addition, we emphasised that the favourable thermal properties of annelated triazinylacetohydrazides studied may enhance their possible utility as candidates for pharmaceutical applications.

## 2. Results and Discussion

### 2.1. Melting Points Evaluated by the DSC

**Figure 2:**
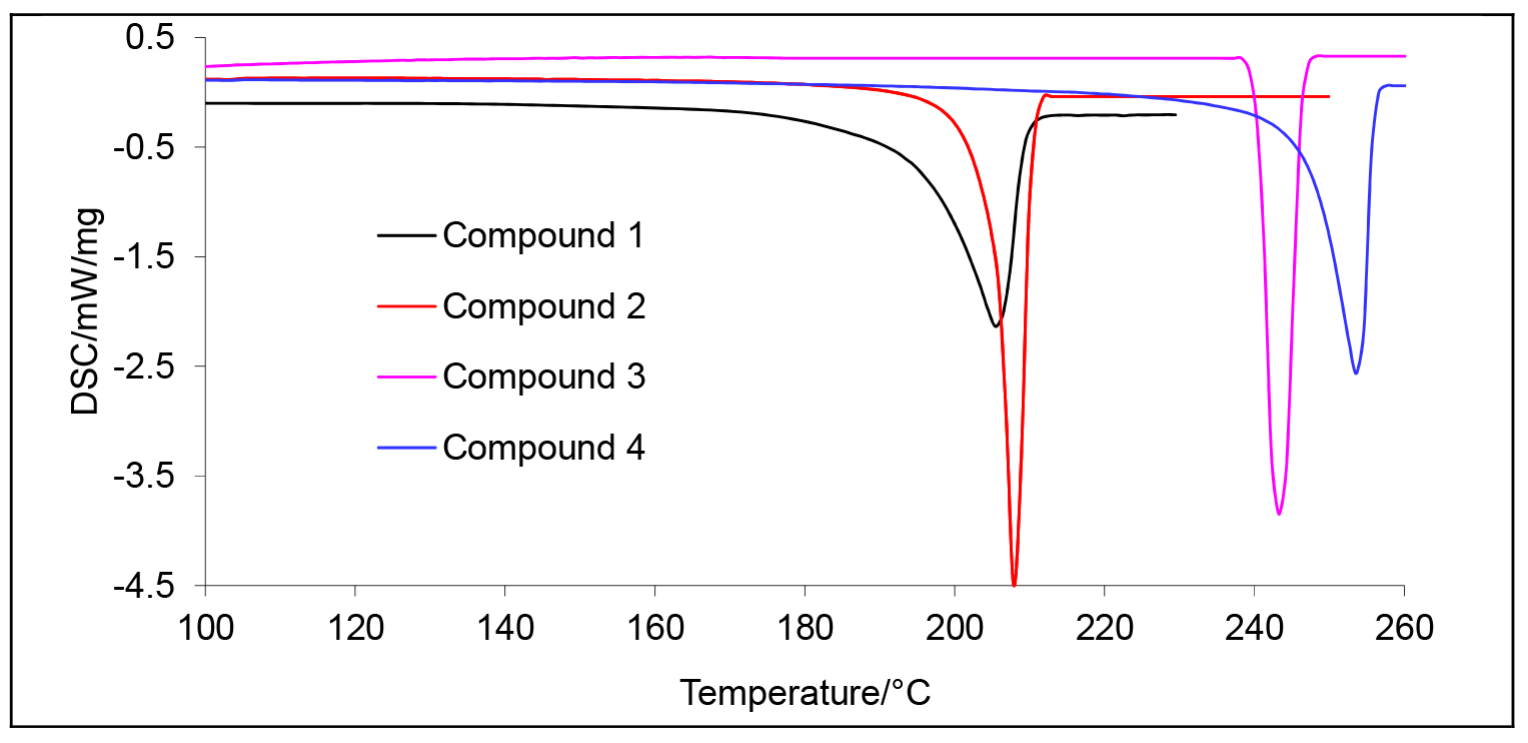
The melting peaks of the tested compounds evaluated with the use of DSC (an argon atmosphere).

The melting peaks for all the tested fused triazinylacetohydrazides evaluated with the use of the DSC method are presented in Figure 2. In turn, the values of the onset (*T*_onset_), maximum (*T*_max_) melting temperatures and the melting enthalpies (Δ*H*) are placed in Table 1. The presented results show that all the investigated molecules have only one endothermic DSC peak related to the melting process and do not undergo polymorphic transformations. In the case of compound **1**, without a substituent at the phenyl moiety (i.e., the parent structure), and the molecule with a *para*-ethoxy group as a substituent (compound **4**), one narrow melting DSC peak is observed. However, for compounds **2** and **3**, bearing the *para*-methyl and *para*-methoxy substituent, respectively, at the same moiety, one DSC peak is also visible, but with a small tail at the beginning of the melting.

The DSC analysis shows that all tested annelated triazinylacetohydrazides are characterised by high melting points (above 195 °C). When comparing the obtained melting points, a certain relationship is visible. As the number of electrons in the molecule increases, the melting point also increases. It has been recently established (based on the developed model suitable for a fast crude estimation of melting point) that the number of electrons in a molecule is directly correlated with the molecular weight. Molecules with a higher number of electrons are able to create more polar dipoles (higher dispersion forces), and, as a result, they have higher melting points [19]. The compound bearing an unsubstituted phenyl moiety (**1**) and its derivative with the *para*-CH_3_ substitution at the phenyl ring (**2**) show melting points with *T*_max_ at 205 °C and 208 °C, respectively. The *para*-OCH_3_ derivative (**3**) has a melting point approx. 40 °C higher than that of the parent structure (**1**). However, the derivative containing a *para*-OC_2_H_5_-substituted phenyl ring (**4**) shows a melting point that is about 50 °C higher than that of unsubstituted structure **1**.

Moreover, as can be seen from Table 1, all the *para*-substituted fused triazinylacetohydrazides (**2**–**4**) show higher melting enthalpies (Δ*H*) as compared to an unsubstituted compound (**1**). The presence of a substituent at the *para* position of the phenyl moiety causes an increase in the Δ*H* values from 12.5 J/g (compound **4**) to 65.9 J/g (compound **2**). These results confirm that more energy must be supplied in the form of heat to change 1 g of the tested compound from a solid to a liquid in the case of all *para*-substituted molecules compared to unsubstituted structure **1** [20,21]. Among compounds with the *para* substitution (**2**–**4**), the dependence between Δ*H* values and the mass of the substituent is also noticed. As the mass of a substituent increases, the melting enthalpy decreases. This indicates that it is easier to melt derivatives containing an alkoxy group than a derivative bearing an alkyl group at the phenyl moiety.

### 2.2. TG/DTG Analyses (Inert Conditions)

**Figure 3:**
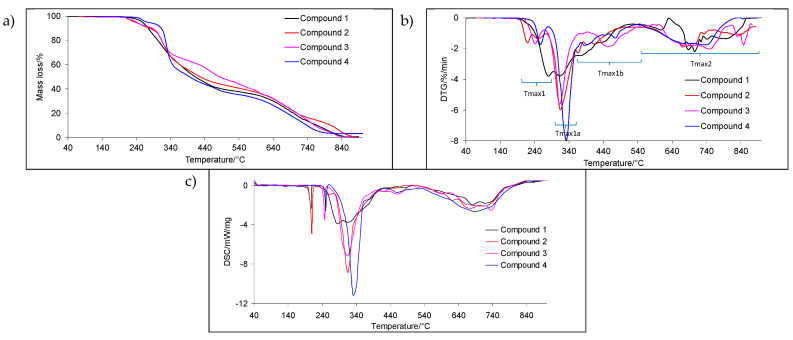
The course of the TG (**a**), DTG (**b**) and DSC (**c**) curves for the tested compounds (inert conditions).

The course of the TG and DTG curves for the tested annelated triazinylacetohydrazides gathered in inert conditions is presented in Figure 3. In addition, the course of the DSC curves collected together with the TG/DTG analysis using a DSC/TG Cp S sample carrier is shown in Figure 3. Table 2 shows the results read from the TG and DTG curves, such as the values of the initial decomposition temperatures (*T*_5%_), peak maximum temperatures (*T*_max_), mass losses (Δ*m*) and the residual mass at 900 °C (rm).

The results from the TG and DTG analyses show that the tested molecules are highly thermally resistant. Their thermal decomposition begins at temperatures above 238 °C. Compounds **2** and **3**, containing the *para*-methyl and *para*-methoxy group, respectively, are characterised by similar thermal stability, and have the lowest thermal stability among all the tested heterobicyclic acetohydrazides (**1**–**4**). Their thermal decomposition starts around 240 °C in inert conditions. However, the highest thermal stability among all the investigated molecules is demonstrated by compound **4**, containing a *para*-ethoxy group at the phenyl moiety. Its thermal resistance is above 270 °C in inert conditions.

As is well visible from the presented TG/DTG curves, the decomposition process of the tested compounds is complex. It includes many simultaneous pyrolysis processes of the bonds present in the structure of the investigated molecules, which is visible in many poorly separated signals on the DTG curve. In general, it can be said that decomposition in an inert atmosphere occurs in two main stages. The first decomposition stage extends over a wide temperature range, namely, from temperature *T*_5%_ to approximately 540 °C. This decomposition stage is composed of three main steps, visible at *T*_max_, *T*_max1a_ and *T*_max1b_. The first step (*T*_max_) appears up to a temperature of 300 °C and is described by one or two DTG signals that are not well separated. The second step (*T*_max1a_) spreads from 300 °C to 360–370 °C. However, the third step (*T*_max1b_) happens between 360–370 °C and 550 °C, and it is composed of two main DTG signals with a low intensity that are not well separated. The mass loss (Δ*m*_1_) is significant during the first decomposition stage. It is from 59.5% to 65%. The second decomposition stage takes place above the temperature of 540 °C, and it is also composed of at least three or four steps. The mass loss is from 31.8% to 40.5% in the temperature range of 540–900 °C. Basically, all the compounds decompose completely when heated to 900 °C; only a slight residue at 900 °C (0.0–3.2%) is observed. The DSC analysis also confirms that the decomposition process of the tested compounds can be divided into two main stages consisting of several steps. The first asymmetric DSC decomposition peak is visible between the temperatures of 240 °C and 550 °C. This DSC peak is an endothermic signal, which indicates the pyrolysis reactions of the bonds present in the structure of the tested compounds. The second DSC peak as a broad signal at temperatures above 550 °C is visible. It is also connected with the pyrolysis processes (an endothermic signal). In addition, the comparison of the TG/DTG and DSC results performed under the same experimental conditions indicates that the tested compounds may undergo partial decomposition immediately after their melting or during melting. This is confirmed by the presence of a low-intensity DTG signal at temperatures slightly above or equal to the melting temperatures defined as *T*_max_ from the DSC analysis.

### 2.3. Decomposition Course of the Tested Compounds Evaluated with the Use of Simultaneous TG/FTIR/QMS Method (Inert Conditions)

**Figure 4:**
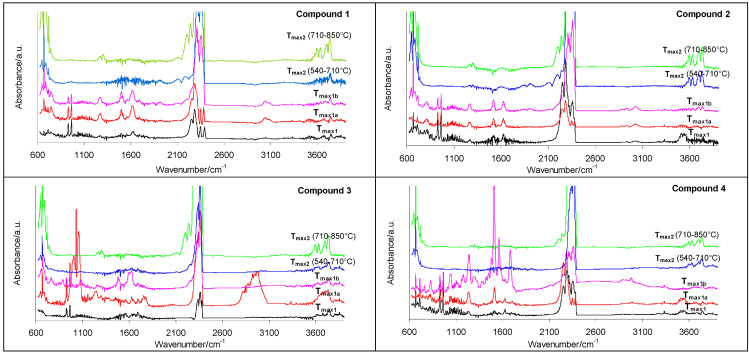
The gaseous FTIR spectra collected at *T*_max1_, *T*_max1a_, *T*_max1b_ and *T*_max2_ (inert conditions).

The gaseous FTIR spectra obtained during heating of the analysed compounds and collected at *T*_max1_, *T*_max1a_, *T*_max1b_ and *T*_max2_ in inert conditions are presented in Figure 4. At *T*_max1_, there is the attendance of two absorption peaks at 931 cm^−1^ and at 966 cm^−1^ that are characteristic bands for the emission of NH_3_ (deformation vibrations of N–H groups [22,23,24,25]). The formation of HNCO by the presence of the absorption peaks at 2270–2290 cm^−1^ [22,26,27] is confirmed. The beginning of HCN emission (the absorption band at 713 cm^−1^ [28,29]) and volatile compounds containing the carbonyl group in their structure (the stretching vibrations of C=O at 1690–1750 cm^−1^) at this temperature are also visible. In addition, the attendance of the absorption bands at 708–742 cm^−1^ (the out-of-plane deformation vibrations of the N–H and the C_Ar-H_), 1230–1282 cm^−1^ (the stretching vibrations of the C–N), 1492–1560 cm^−1^ (the stretching vibrations of the C_Ar_=C_Ar_), 1619–1690 cm^−1^ (the deformation vibrations of the N–H), 2856–2913 cm^−1^ (the stretching vibrations of the C–H) and 3008–3064 cm^−1^ (the stretching vibrations of the C_Ar_-_H_) indicates the formation of some aromatic compounds with amine groups [22,30,31]. In turn, the absorption band at 3332 cm^−1^ may confirm the creation of some compounds containing the -NHCO group in the structure. Some of the volatiles such as CO_2_ (the absorption bands at 2300–2365 cm^−1^ and 669 cm^−1^) and H_2_O (the absorption bands of OH above 3500 cm^−1^) are also created. As the temperature increases to *T*_max1a_, the intensity of the previously mentioned volatiles increases. Their evaporation up to the temperature of 540 °C is clearly visible.

**Figure 5:**
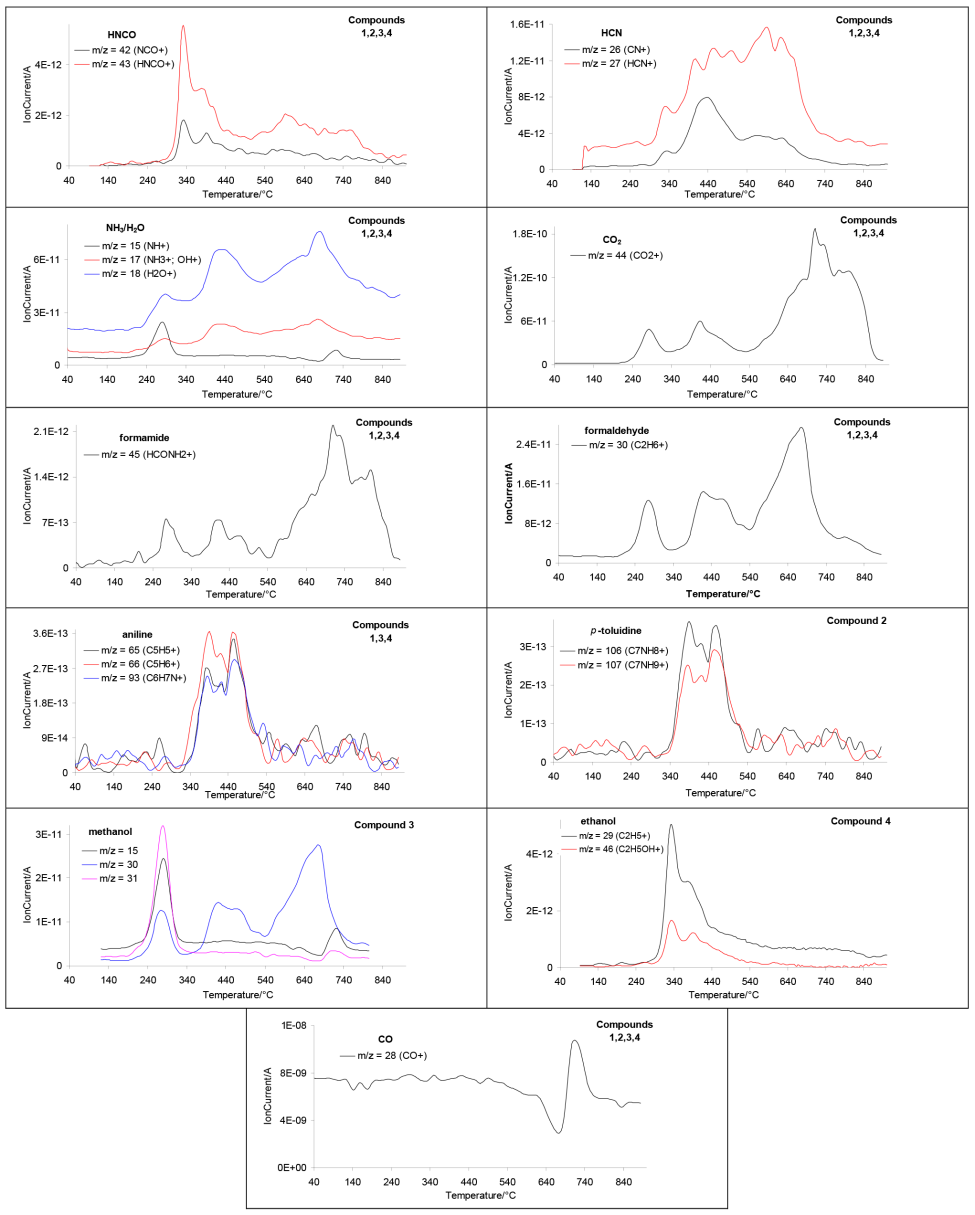
The exemplary QMS spectra of volatiles formed during pyrolysis of the tested compounds.

If you look at the results obtained from the QMS analysis, Figure 5, it can be concluded that, in the temperature range of *T*_5%_–540 °C, the emission of isocyanic acid—HNCO (*m*/*z* ions: 43 (HNCO^+^), 42 (NCO^+^)), hydrogen cyanide—HCN (*m*/*z* ions: 27 (HCN^+^) and 26 (CN^+^)), ammonia—NH_3_ (*m*/*z* ions: 17 (NH_3_^+^), 15 (NH^+^)), formamide—HCONH_2_ (*m*/*z* ion: 45 (HCONH_2_^+^)), formaldehyde—H_2_CO (*m*/*z* ion: 30 (H_2_CO^+^), CO_2_ (*m*/*z* ion: 44 (CO_2_^+^) and H_2_O (*m*/*z* ions: 18 (H_2_O^+^), 17 (HO^+^)) is completely confirmed. Apart from the emission of these gases, from a temperature of approx. 305 °C to a maximum at *T*_max1b_, the formation of aromatic compounds is observed. The creation of aniline as a result of pyrolysis of unsubstituted compound **1** by the presence of *m*/*z* ions 93 (C_6_H_7_N^+^), 65 (C_5_H_5_^+^) and 66 (C_5_H_6_^+^) is confirmed [32]. In the case of compound **2**, containing the *para*-CH_3_-substituted phenyl moiety, the formation of *para*-toluidine *m*/*z* ions 107 (C_7_NH_9_^+^) and 106 (C_7_NH_8_^+^) is proven. However, for compound **3**, with the *para*-OCH_3_ group, interesting observations from the FTIR and QMS spectra are found. At *T*_max1a_, the absorption bands at 1002, 1031 and 1060 cm^−1^ (the stretching vibrations of the C–O), at 1195–1230 cm^−1^ (the deformation vibrations of the C–H), at 2817–2993 cm^−1^ (the stretching vibrations of the C–H) and above 3550 cm^−1^ (the stretching vibrations of the OH) and the presence of *m*/*z* ions 31 (CH_3_O^+^), 15 (CH_3_^+^) and 30 (CH_2_O^+^) confirm the creation of methanol as a one of the volatiles, which suggests the detachment of the -OCH_3_ group from the aromatic benzene ring during the pyrolysis process. As a result, as an aromatic volatile, mainly the formation of aniline is observed. A similar situation occurs in the case of pyrolysis of the *para*-substituted compound with the -OC_2_H_5_ group (**4**). The absorption bands at 1054–1066 cm^−1^ and 1229–1282 cm^−1^ (the stretching vibrations of the C–O), at 1382–1407 cm^−1^ (the deformation vibrations of the C–H), 2914–2967 cm^−1^ (the stretching vibrations of the C–H) and above 3500 cm^−1^ (the stretching vibrations of the OH) and the *m*/*z* ions 45 (C_2_H_5_O^+^), 31 (CH_2_OH^+^) and 29 (C_2_H_5_^+^) indicate the formation of ethanol as a result of the loss of the -OC_2_H_5_ group during pyrolysis and aniline as an aromatic decomposition product [33,34,35].

**Scheme 1:**
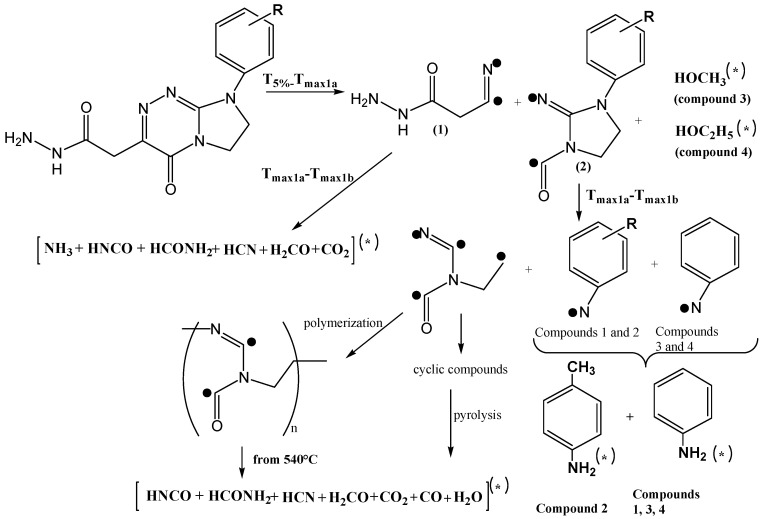
The simplified scheme of the pyrolysis course for the tested compounds. (*)—the gaseous products experimentally confirmed based on the FTIR and QMS results. Compounds: **1**: R = H; **2**: R = 4-CH_3_; **3**: R = 4-OCH_3_; **4**: R = 4-OC_2_H_5_.

Taking into account the experimental and theoretical mass loss in the first two decomposition steps (between *T*_5%_ and *T*_max1a_), Table 3, the molar mass of the tested compounds and the type of the emitted volatiles, it can be assumed that, in these decomposition steps, mainly the symmetric cleavage of N–N and N–C bonds takes place, combined with the detachment of fragments containing the 2-cyanoacetohydrazide diradical (H_2_N-NH-CO-CH_2_-C^•^=N^•^)—marked in Scheme 1 as a diradical (1)—which decomposes into volatiles with smaller molecular masses. In the case of compounds **3** and **4**, in this temperature range, the detachment of an alkoxy group from the aromatic ring also occurs, which results in the emission of an appropriate alcohol molecule. In turn, the remaining part of the compound (marked in Scheme 1 as a diradical (2)) decomposes with the emission of compounds containing an aromatic ring in their structure in the temperature range of *T*_max1a_–*T*_max1b_. It should be remembered that the pyrolysis of the tested compounds takes place according to the radical mechanism, and, therefore, various intermediate reactions between radicals are possible. This generates the continued emission of gaseous products such as NH_3_, HCN, HNCO, HCONH_2_, H_2_CO, CO_2_ and H_2_O, as is confirmed based on the FTIR and QMS results, Figure 4 and Figure 5.

At the second decomposition stage (from a temperature of approx. 540 °C), mainly the emission of HCN (the absorption band at 713 cm^−1^ and *m*/*z*: 27 (HCN^+^) and 26 (CN^+^)), HNCO (the absorption bands at 2270–2290 cm^−1^ and *m*/*z*: 43 (HNCO^+^), 42 (NCO^+^)), CH_3_CN (the absorption bands at 2200–2237 cm^−1^ and *m*/*z*: 41 (CH_3_CN^+^), 40 (CH_2_CN^+^), 39 (CHCN^+^)), CO_2_ (the absorption bands at 2300–2365 cm^−1^ and 669 cm^−1^ and *m*/*z*: 44 (CO_2_^+^), H_2_O (the absorption bands above 3500 cm^−1^ and *m*/*z*: 18 (H_2_O^+^), 17 (HO^+^)), CO (*m*/*z*: 28 (CO^+^)), HCONH_2_ (*m*/*z*: 45 (HCONH_2_^+^)) and H_2_CO (*m*/*z*: 30 (H_2_CO^+^) is observed [32]. The type of gaseous decomposition products at the second decomposition stage suggests the pyrolysis of the remaining adduct formed from the residue after the removal of the H_2_N-NH-CO-CH_2_-C^•^=N^•^ and an aromatic group from the initial compounds (an adduct formed in the polymerisation process) or the pyrolysis process of the formed cyclic compounds, as is presented in Scheme 1.

### 2.4. TG/DTG Analyses (Oxidative Conditions)

**Figure 6:**
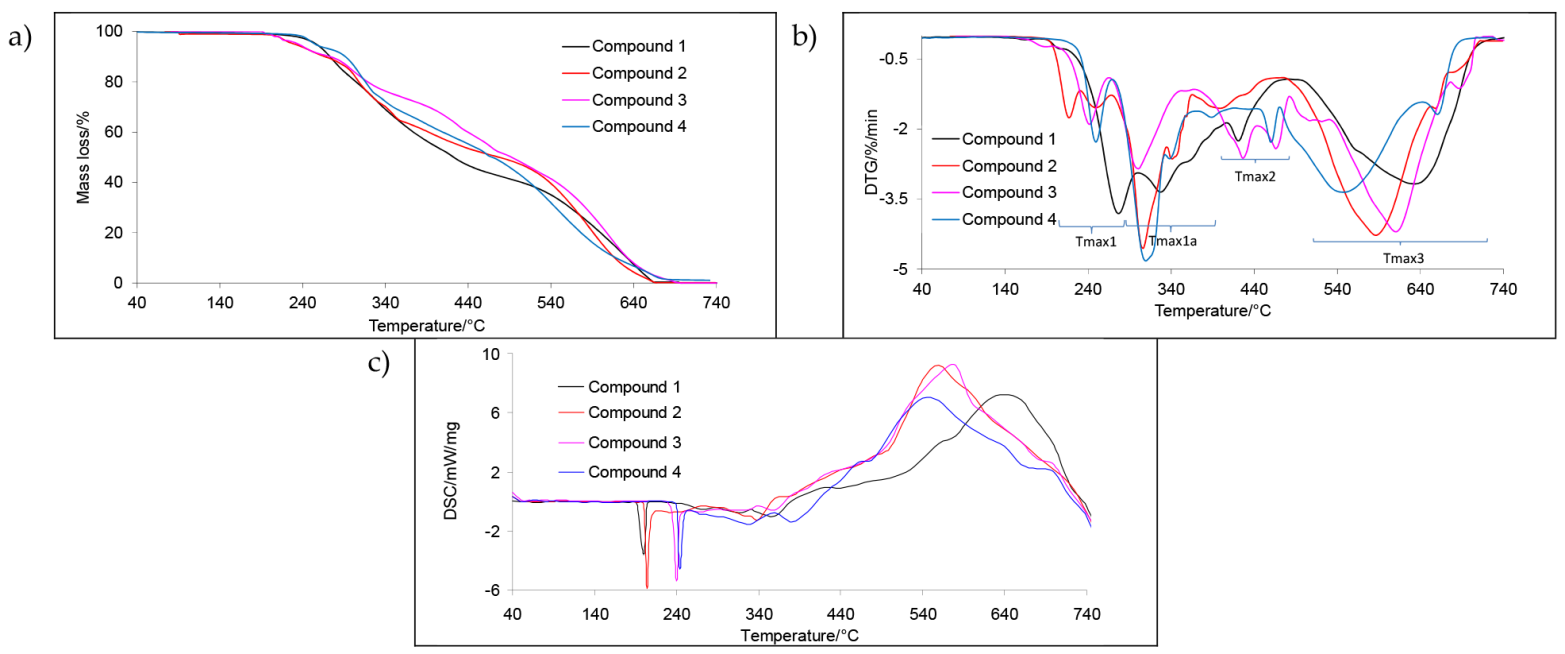
The course of the TG (**a**), DTG (**b**) and DSC (**c**) curves for the tested compounds (oxidative conditions).

The course of the TG, DTG and DSC curves for the tested compounds collected in oxidative conditions is presented in Figure 6. Table 4 shows the results read from the TG and DTG curves. This analysis confirms a high thermal stability of the investigated molecules in the presence of oxygen. All the compounds start to decompose at temperatures higher than 230 °C, i.e., at temperatures significantly above room temperature. In light of the results of studies on the kinetics of thermal decomposition, storing highly thermally stable pharmaceuticals at temperatures from 25 to 40 °C under air atmosphere is not associated with the risk of losing their shelf life [36,37]. Among all annelated triazinylacetohydrazides, compound **4**, containing the *para*-ethoxy group at the phenyl moiety, is the most thermally stable. Its decomposition begins at 254 °C. The analysed compounds are less thermally resistant in an oxidising atmosphere compared to in an inert atmosphere. However, this is an expected situation because oxygen interacts with these compounds at elevated temperatures, resulting in a decrease in decomposition activation energy. Similarly to in the inert atmosphere, TG/DTG results indicate that the decomposition of the investigated molecules in the presence of air is a complicated process in which individual elementary reactions overlap. In general, it can be said that the tested compounds decompose in three main stages under oxidising conditions, marked as *T*_max1_/*T*_max1a_, *T*_max2_ and *T*_max3_. The first decomposition stage is composed of few steps. It starts from *T*_5%_ and ends at temperatures of approx. 380–410 °C. The mass loss is from 27% to 47%. The second decomposition stage from approx. 380–410 °C to approx. 470–480 °C, with the mass loss from 10% to 21%, is visible. Finally, the third decomposition stage spreads from 470–480 °C to 740 °C with the mass loss above 42%. All analysed compounds, when heated to 750 °C, decompose completely in an oxidising atmosphere. Due to the presence of oxygen, it is expected that the decomposition process will be more complicated than in an atmosphere without oxygen, which will result in a greater variety of gaseous decomposition products. These results are also confirmed by the DSC analysis. As is well seen, both endothermic and exothermic signals are observed on the DSC curves, Figure 6. The beginning of the first decomposition stage of the tested compounds is related to the occurrence of endothermic DSC signals, which describe the process of breaking the bonds present in their structure. As the heating temperature increases, the appearance of exothermic DSC peaks is visible. It confirms that the decomposition of the investigated compounds is more complicated in an air atmosphere. It includes both pyrolysis processes and reactions with oxygen.

### 2.5. Decomposition Course of the Tested Compounds Evaluated with the Use of Simultaneous TG/FTIR/QMS Method (Oxidative Conditions)

**Figure 7:**
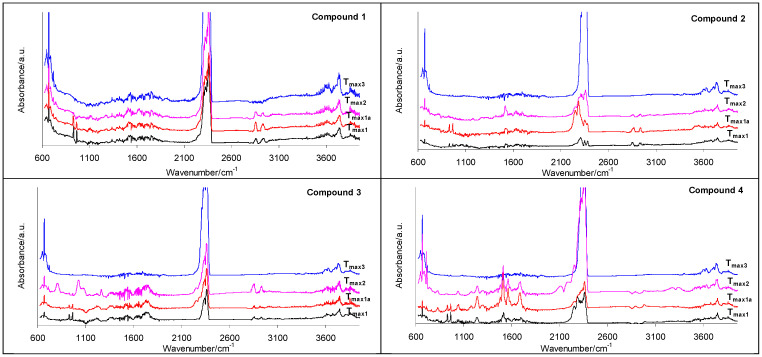
The gaseous FTIR spectra collected at *T*_max1_, *T*_max1a_, *T*_max2_ and *T*_max3_ (oxidative conditions).

Figure 7 shows the gaseous FTIR spectra obtained during heating of the tested annelated triazinylacetohydrazides and collected at *T*_max1_, *T*_max1a_, *T*_max2_ and *T*_max3_ in oxidative conditions.

As is well visible, from *T*_5%_ to *T*_max1a_, the emission of NH_3_ due to the presence of two characteristic absorption bands at 931 cm^−1^ and at 966 cm^−1^, describing the deformation vibrations of N–H groups [22,23,24,25] at the gaseous FTIR spectra, and *m*/*z* ions 15 (NH^+^) and 17 (NH_3_^+^) at the QMS spectra is clearly confirmed. Together with the ammonia, the creation of some of the nitrogen oxides is documented. The emission of NO_2_ as a result of oxidation of ammonia [38,39,40] extends from *T*_5%_ to 490 °C (the first low emission). The second, high emission of nitrogen oxides with a maximum intensity occurs at *T*_max3_. The formation of these nitrogen oxides is confirmed by the *m*/*z* ion 30 (NO^+^) at the QMS spectra and the presence of the absorption bands at 1630 cm^−1^ at the FTIR spectra. Also, the emission of HNCO starts at *T*_5%_, and it is well visible up to temperatures of 800 °C with two clearly marked maxima at *T*_max1a_ and *T*_max3_. This emission is illustrated by the presence of absorption bands at 2270–2290 cm^−1^, partially obscured by absorption bands for CO_2_ and *m*/*z* ions 43 (HNCO^+^) and 42 (NCO^+^). However, the emission of HCN (FTIR: the absorption signal at 713 cm^−1^; *m*/*z*: 27 (HCN^+^) and 26 (CN^+^)) from ca. *T*_max2_ is recorded. Together with the emission of the above-mentioned gases, the emission of CO_2_ (*m*/*z*: 44 (CO_2_^+^) and H_2_O (FTIR: *m*/*z* ions: 18 (H_2_O^+^), 17 (HO^+^)) is observed.

It is noteworthy that no alcohol formation as a result of the detachment of alkoxy groups from the aromatic ring in the case of compounds **3** and **4** under oxidising conditions is observed. This confirms that alcohols (methanol and ethanol) as intermediate products are oxidised to aldehydes (formaldehyde and acetaldehyde) [41,42,43]. The formed aldehydes can be further oxidised to the corresponding acids that may undergo decarboxylation under the conditions of the experiment [44,45,46]. The absorption bands at 1710–1745 cm^−1^ confirm the creation of some carbonyl-type compounds as a result of the cleavage of the bonds present in the structure of the tested compounds (formamide, formaldehyde) and as a result of the oxidation of alcohols (formaldehyde and acetaldehyde). In addition, the QMS spectra prove the creation of aldehyde fragments by the presence of the characteristic *m*/*z* ions: 29 (CHO^+^), 30 (H_2_CO^+^), 44 (CH_3_CHO^+^) and 45 (HCONH_2_^+^).

From the temperature of *T*_max1a_ to *T*_max2_, the clear emission of some aromatic compounds, based on the presence of the absorption bands at 700–815 cm^−1^ (the out-of-plane deformation vibrations of the N–H and the C_Ar-H_), 1234–1278 cm^−1^ (the stretching vibrations of the C–N), 1500–1560 cm^−1^ (the stretching vibrations of the C_Ar_=C_Ar_), 1619–1675 cm^−1^ (the deformation vibrations of the N–H), 2842–2925 cm^−1^ (the stretching vibrations of the C–H) and 3000–3070 cm^−1^ (the stretching vibrations of the C_Ar-H_), is visible [22,30,31]. Their intensity is lower as compared to their emission in inert conditions, which may indicate their partial combustion in the presence of air atmosphere [47,48].

**Figure 8:**
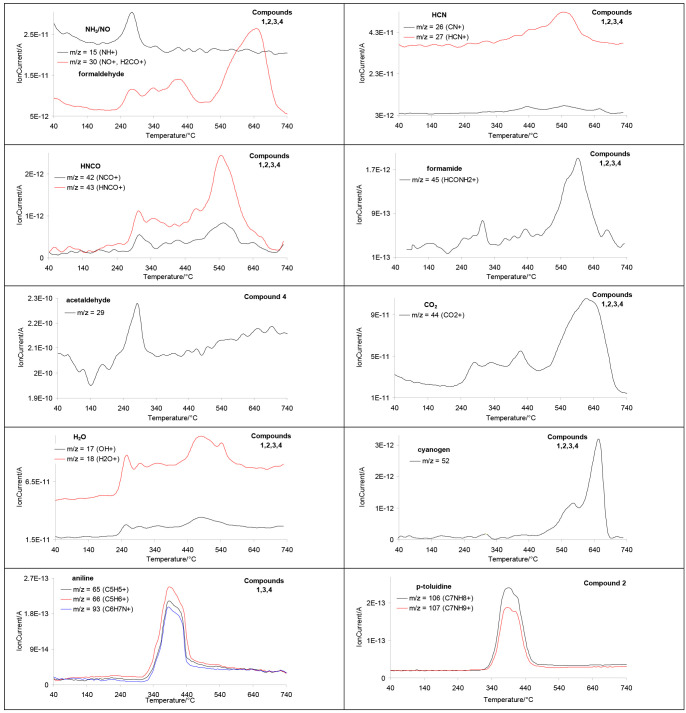
The exemplary QMS spectra of volatiles formed during oxidative decomposition of the tested compounds.

Above 500 °C, with the maximum at ca. 550 °C, the evaporation of HNCO, HCN, HCONH_2_ and H_2_O is clearly visible from the FTIR and QMS spectra, Figure 7 and Figure 8. When the temperature increases above 550 °C, the formation of the gaseous decomposition products such as NO_2_ (*m*/*z* 30), CO_2_ (*m*/*z* 44) and cyanogen (C_2_N_2_, *m*/*z* 52) [49,50,51], with the maximum intensity at ca. 640 °C, is observed.

**Scheme 2:**
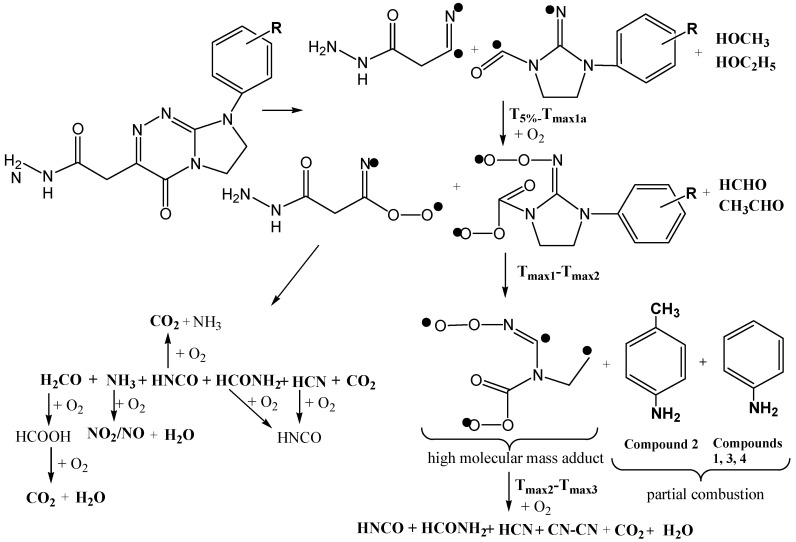
The simplified oxidising decomposition course of the tested compounds. Compounds: **1**: R = H; **2**: R = 4-CH_3_; **3**: R = 4-OCH_3_; **4**: R = 4-OC_2_H_5_.

The obtained results indicate the reactions between gaseous decomposition products resulting from the breaking of the bonds present in the structure of the tested compounds and oxygen. In particular, these are reactions between ammonia and oxygen leading to nitrogen oxides and water. It is also worth mentioning the reaction between hydrogen cyanide and oxygen, which leads to isocyanic acid and cyanogen being obtained at higher temperatures [52,53,54]. The maximum emission of HNCO above 500 °C is visible. In turn, the maximum emission of HNCO at ca. 340–350 °C in an inert atmosphere is confirmed. However, the cyanogen is emitted at temperatures above 550 °C, as is proved based on the performed analyses. Moreover, no alcohol emission and lower emission of aromatic compounds confirm the oxidation and combustion reactions of volatiles. A simplified diagram of possible reactions when heating the tested compounds in the presence of oxidising atmosphere is given in Scheme 2. This scheme presents only a very simplified reaction course. In the case of decomposition in the presence of oxygen, many simultaneous radical reactions between volatiles and oxygen in a gas phase are highly expected. They lead to uncontrolled radical reactions, during which various derivatives with smaller and higher molecular masses can be created and undergo oxidative decomposition and combustion.

## 3. Materials and Methods

### 3.1. Differential Scanning Calorimetry (DSC)

The DSC method (a DSC 204, Netzsch, Selb, Germany) was applied in order to evaluate the course of the melting of the tested compounds. The samples were heated from 20 °C to 260 °C in aluminium crucibles with a pierced lid with a heating rate of 10 K min^−1^ and in argon atmosphere (a flow rate of 40 mL min^−1^). The onset (*T*_onset_), peak maximum (*T*_max_) melting temperatures and the melting enthalpy (Δ*H*) were determined from the obtained DSC curves.

### 3.2. Simultaneous Thermogravimetric Analysis Coupled on-Line with FTIR and QMS Analysers (TG/DTG/FTIR/QMS)

Simultaneous TG/DTG analysis (a STA 449 Jupiter F1 instrument Netzsch, Selb, Germany) coupled on-line with FTIR and QMS analysers to estimate thermal properties, such as the thermal stability, decomposition stages and mass losses, as well as the course of pyrolysis and oxidative decomposition, was used. The tested samples from 40 °C to 900 °C in open Al_2_O_3_ crucibles with a heating rate of 10 K min^−1^ and in helium (a flow rate of 40 mL min^−1^) and in synthetic air (a flow rate of 100 mL min^−1^) were heated. The DSC/TG Cp S sample carrier was used. The 5% of mass loss (*T*_5%_), maximum decomposition temperatures (*T*_max_) and mass loss (Δ*m*) were estimated.

FTIR TGA 585 r (Bruker, Mannheim, Germany) and a QMS 403 C Aëolos (Netzsch, Selb, Germany) analysers were used to evaluate the type of emitted volatiles in both furnace atmospheres. An FTIR TGA 585 analyser with an IR cell heated to 200 °C was connected on-line to an STA instrument by a Teflon transfer line with a diameter of 2 mm heated to 200 °C. The gaseous FTIR spectra in the wavenumber range of 600–4000 cm^−1^ and with a resolution of 4 cm^−1^ and 32 scans per spectrum were collected. A QMS analyser operated under electron ionisation of 70 eV was connected on-line to an STA instrument by quartz capillary heated to 300 °C. The formed *m*/*z* ions were collected from 10 amu to 150 amu. In each heating cycle, the QMS spectra at a time interval of 0.6 min (every 6.1 °C) were collected.

## 4. Conclusions

The DSC and TG/DTG/FTIR/QMS methods have been applied to evaluate the thermal stability and course of the melting process, pyrolysis and oxidative decomposition for annelated triazinylacetic acid hydrazides with prospective medical use. Compounds which are unsubstituted and substituted at the *para* position of the phenyl moiety were subjected to detailed thermal studies. It was found that melting points, melting enthalpy values and thermal stabilities of the tested compounds changed depending on the type of substituent. Molecules with an alkoxy group (-OC_2_H_5_ or -OCH_3_) were characterised by the highest melting points, with *T*_max_ at 254 °C and 243 °C, respectively. In turn, the unsubstituted compound and its derivative containing the *para*-CH_3_-substituted phenyl ring showed the lowest melting points with *T*_max_ at 205 °C and 208 °C, respectively. The change in their thermal stability depending on the used atmosphere was analogical. The highest thermal stability (in both atmospheres) revealed the compound substituted by the *para*-OC_2_H_5_ group. It was thermally stable up to 272 °C under inert conditions and up to 254 °C under oxidative conditions. In turn, the lowest thermal resistance was observed for the compound bearing the *para*-CH_3_ group (238 °C and 230 °C in inert and oxidative conditions, respectively). The obtained results clearly indicated that all the analysed annelated triazinylacetohydrazides in the experimental conditions used revealed the enthalpy values necessary for their melting in the range of 209.4–275.3 J/g, did not undergo polymorphic transformations during their melting and proved to be materials with high thermal stability. Thus, when assessing the therapeutic usefulness of these potential drugs, their high thermal stability and favourable thermal properties should be taken into account. In both atmospheres, the decomposition process of annelated triazinylacetohydrazides included several stages that were not well divided. The identified volatiles with the use of the simultaneous TG/FTIR/QMS analyses indicated that the pyrolysis and oxidative decomposition proceeded by a radical mechanism. In inert conditions, the formation of NH_3_, HCN, HNCO, HCONH_2_, HCHO, CO_2_, CO and H_2_O (for all the tested compounds) and *para*-toluidine (for the compound with the *para*-CH_3_ substitution) and the emissions of aniline and alcohol such as methanol or ethanol (for compounds bearing *para*-OCH_3_ and *para*-OC_2_H_5_ substitutions) were confirmed. This indicated the cleavage of C–N and N–N bonds in the triazine ring and resulted in the formation of the radical fragments. These radicals reacted with each other and broke down into fragments with lower molecular masses, detected by the FTIR and QMS analysers. In turn, in oxidative conditions, the mixture of volatiles NH_3_, HCN, NO, HNCO, HCONH_2_, CO_2_, H_2_O and cyanogen (for all the compounds), *para*-toluidine (for the compound bearing the *para*-CH_3_ substituent), aniline (for unsubstituted, *para*-OCH_3_ and *para*-OC_2_H_5_ substituted compounds) and acetaldehyde (for the compound with the *para*-OC_2_H_5_ group) was clearly observed. The additional release of nitrogen oxides and cyanogen and the lack of the emission of alcohols proved the reactions of initially formed volatiles (in particular, NH_3_, HCN, HNCO and alcohols) with oxygen and thus the more complicated mechanism of oxidative decomposition of the tested compounds compared to their pyrolysis in inert conditions.

We believe that this study will contribute to the understanding of the thermal decomposition mechanism and course of these pharmacologically important heterocyclic molecules and their thermal properties. We hope that the results obtained will be of practical importance, as they will enable determination of the optimal storage conditions for these potential pharmaceuticals and will allow application of filters capturing volatile degradants generated during their thermal disposal.

## Figures and Tables

**Table 1:** The onset (*T*_onset_), maximum (*T*_max_) melting temperatures and the melting enthalpy values (Δ*H*) for the tested compounds evaluated with the use of DSC.

Compound	M (g/mol)	*T*_onset_ (°C)	*T*_max_ (°C)	Δ*H* (J/g)
**1**	286	195	205	209.4
**2**	300	206	208	275.3
**3**	316	241	243	266.5
**4**	330	247	254	221.9

**Table 2:** The TG/DTG data for the tested compounds (inert conditions).

Compound	Decomposition Process							
	First Decomposition Stage					Second Decomposition Stage		
	*T*_5%_ (°C)	*T*_max1_ (°C)	*T*_max1a_ (°C)	*T*_max1b_ (°C)	Δ*m*_1_ (%)	*T*_max2_ (°C)	Δ*m*_2_ (%)	rm (%)
**1**	260	279	314	379/444	61.4	689/709/744/794	37.8	0.8
**2**	238	221/253	316	368/415	59.5	686/710/736/826	40.5	0.0
**3**	240	245	316	385/461	63.4	673/758/835/848	35.9	0.7
**4**	272	257	332	392/477	65.0	672/742/852	31.8	3.2

**Table 3:** The experimental (Δ*m_exp_*) and theoretical (Δ*m_theor_*) mass loss for the tested compounds.

Compound	Decomposition Process							
	First Decomposition Stage						Second Decomposition Stage	
	Δ*m*_1*a*_ *(T*_5%_–*T*_max1a_) (%)		Δ*m*_1*b*_ *(T*_max1a_–*T*_max1b_) (%)		Δ*m*_1_*a* + Δ*m*_1*b*_ (%)		Δ*m*_2_ (%) (540–850 °C)	
	Δ*m*_1*exp*_	Δ*m*_1*theor*_	Δ*m*_1*exp*_	Δ*m*_1*theor*_	Δ*m*_1*exp*_	Δ*m*_1*theor*_	Δ*m*_1_*_exp_*	Δ*m*_1*theor*_
**1**	37.0	34.6	24.4	32.1	61.4	66.7	37.8	33.3
**2**	36.5	33.0	23.0	31.6	59.5	64.6	40.5	35.4
**3**	34.5	31.3	28.9	37.6	63.4	68.9	35.9	31.1
**4**	44.0	30.0	21.0	40.3	65.0	70.3	31.8	29.7

**Table 4:** The TG/DTG data for the tested compounds (oxidising conditions).

Compound	Decomposition Process							
	First Decomposition Stage				Second Decomposition Stage		Third Decomposition Stage	
	*T*_5%_ (°C)	*T*_max1_ (°C)	*T*_max1a_ (°C)	Δ*m*_1_ (%)	*T*_max2_ (°C)	Δ*m*_2_ (%)	*T*_max3_ (°C)	Δ*m*_3_ (%)
**1**	250	211/276	327/364	46.7	416	11.1	559/631	42.2
**2**	230	218/241	304/339	35.9	401/433	13.6	586/657	50.5
**3**	230	186/244	301/361	27.4	426/466	21.0	506/606/691	51.6
**4**	254	249	309/336/389	40.4	459	9.7	549/662	49.9

## Data Availability

The data presented in this study are available on request from the authors.
